# Consumer Willingness to Share Personal Digital Information for Health-Related Uses

**DOI:** 10.1001/jamanetworkopen.2021.44787

**Published:** 2022-01-24

**Authors:** David Grande, Nandita Mitra, Raghuram Iyengar, Raina M. Merchant, David A. Asch, Meghana Sharma, Carolyn C. Cannuscio

**Affiliations:** 1 Division of General Internal Medicine, University of Pennsylvania, Philadelphia; 2 Leonard Davis Institute of Health Economics, University of Pennsylvania, Philadelphia; 3 Department of Biostatistics, Epidemiology and Informatics, University of Pennsylvania, Philadelphia; 4 Wharton School, University of Pennsylvania, Philadelphia; 5 Department of Emergency Medicine, University of Pennsylvania, Philadelphia; 6 Penn Medicine Center for Health Care Innovation, University of Pennsylvania, Philadelphia; 7 Department of Family Medicine and Community Health, University of Pennsylvania, Philadelphia

## Abstract

**Importance:**

Consumers routinely generate digital information that reflects on their health.

**Objective:**

To evaluate the factors associated with consumers’ willingness to share their digital health information for research, health care, and commercial uses.

**Design, Setting, and Participants:**

This national survey with an embedded conjoint experiment recruited US adults from a nationally representative sample, with oversampling of Black and Hispanic panel members. Participants were randomized to 15 scenarios reflecting use cases for consumer digital information from a total of 324 scenarios. Attributes of the conjoint analysis included 3 uses, 3 users, 9 sources of digital information, and 4 relevant health conditions. The survey was conducted from July 10 to 31, 2020.

**Main Outcomes and Measures:**

Participants rated each conjoint profile on a 5-point Likert scale (1-5) measuring their willingness to share their personal digital information (with 5 indicating the most willingness to share). Results reflect mean differences in this scale from a multivariable regression model.

**Results:**

Among 6284 potential participants, 3543 (56%) responded. A total of 1862 participants (53%) were female, 759 (21%) identified as Black, 834 (24%) identified as Hispanic, and 1274 (36%) were 60 years or older. In comparison with information from electronic health care records, participants were less willing to share information about their finances (coefficient, −0.56; 95% CI, −0.62 to −0.50), places they visit from public cameras (coefficient, −0.28; 95% CI, −0.33 to −0.22), communication on social media (coefficient, −0.20; 95% CI −0.26 to −0.15), and their search history from internet search engines (coefficient, −0.11; 95% CI, −0.17 to −0.06). They were more willing to share information about their steps from applications on their phone (coefficient, 0.22; 95% CI, 0.17-0.28). Among the conjoint attributes, the source of information (importance weight: 59.1%) was more important than the user (17.3%), use (12.3%), and health condition (11.3%). Four clusters of consumers emerged from the sample with divergent privacy views. While the context of use was important, these 4 groups expressed differences in their overall willingness to share, with 337 participants classified as never share; 1116 classified as averse to sharing (mean rating, 1.64; 95% CI, 1.62-1.65); 1616 classified as uncertain about sharing (mean rating, 2.84; 95% CI, 2.81-2.86); and 474 classified as agreeable to sharing (mean rating, 4.18; 95% CI, 4.16-4.21). Respondents who identified as White and non-Hispanic, had higher income, and were politically conservative were more likely to be in a cluster that was less willing to share (ie, never or averse clusters).

**Conclusions and Relevance:**

These findings suggest that although consumers’ willingness to share personal digital information for health purposes is associated with the context of use, many have strong underlying privacy views that affect their willingness to share. New protections may be needed to give consumers confidence to be comfortable sharing their personal information.

## Key Points

**Question:**

What factors are associated with consumers’ willingness to share their digital information for health-related uses?

**Findings:**

In this survey study of 3543 US adults, consumer willingness to share digital data was associated with a range of factors, most importantly the source and type of data. Certain data (eg, financial, social media, public cameras) were viewed as more sensitive than electronic health record data, but underlying views on digital health privacy were strongly associated with consumer views on sharing any digital information.

**Meaning:**

In this study, many consumers were reluctant to share their digital data for health-related uses, suggesting that new privacy protections may be needed to increase consumer trust.

## Teaser

This survey study evaluates the factors associated with consumers’ willingness to share their digital health information for research, health care, and commercial uses.

## Introduction

Twenty-five years ago, the Health Insurance Portability and Accountability Act (HIPAA) of 1996 was signed into law.^1^ The privacy provisions were intended to strengthen protections over health information generated in the course of health care delivery.^2^ Since that time, consumer digital information created outside of health care encounters has proliferated, with much of this information reflecting personal health. The proliferation of consumer digital data alongside modern data science and an understanding of health’s social determinants has effaced any lines between health and nonhealth information such that most digital data are now health data.^3^ The result is that the health data generated in the context of clinical care is substantially protected, but information potentially as health-revealing that is collected in other contexts is not.

Although prior studies have demonstrated that consumers care deeply about health privacy,^4,5,6^ views on privacy differ widely based on contextual factors, such as the perceived social benefits of the use, the motive of the user (including if the use is for commercial gain), and the sensitivity of the information itself.^7,8,9^ Digital data has many potential health-related applications. Social media chatter has been mined to identify individuals with mental health concerns that can be directly addressed through outreach.^10,11,12^ Other technologies have been used to suggest improvements to sleep habits, encourage physical activity, and detect falls in the home.^13,14,15,16^ Digital data have also powered responses to the COVID-19 pandemic; mobile devices have been used to track high-risk COVID-19 exposures while also contributing data on risk factors for disease transmission.^10,11^ Alongside these new applications, privacy concerns have grown. A recent analysis found that only 32% of COVID-19 applications available explicitly stated that user data will be anonymized, encrypted, and secured.^17^ There are additional concerns that consumers often cannot turn off the collection of personal data—companies, including search engines and social media sites, can deduce locations of individuals even if users have opted out of sharing location data.^18,19^

These tensions between individual loss of privacy and potential benefits lead to important questions about what determines acceptable use for consumers of their data. We studied a nationally representative population to determine what factors are associated with greater or lesser willingness to share personal digital data related to health.

## Methods

Participants were recruited for this cross-sectional survey study from the web-enabled Ipsos KnowledgePanel. Details regarding participant sampling, recruitment, and survey administration can be found in a prior study published from this survey.^20^ In summary, Ipsos is a probability-based panel that is designed to be representative of the US population, in which participants were recruited using address-based sampling methods.^21^ At the time of recruitment, participants were asked to complete a general informed consent process followed by a core survey profile in which participants self-reported key demographic characteristics including race and ethnicity using the US Census Bureau categories. Black and Hispanic panel members were oversampled for this study to permit subgroup analyses. Race and ethnicity were assessed in this study, as prior evidence demonstrates that in certain circumstances, members of racial and ethnic minority groups are more concerned about their privacy.^22,23^

The survey was administrated between July 10 and July 31, 2020, in Spanish and English. All data received by the study team were deidentified. This study was reviewed and determined exempt by the University of Pennsylvania institutional review board. This study followed the ethical conduct and reporting guidelines of public opinion and survey research defined by the American Association for Public Opinion Research (AAPOR).

### Survey Instrument and Conjoint Scenarios

This study was designed to use conjoint analysis to measure consumer preferences with respect to sharing digital health information. Conjoint analysis has been widely used in marketing and increasingly in health-related applications to measure preferences that more closely reflect revealed interests that may be repeated in other contexts and decisions.^24,25,26^ Conjoint analysis also allows the examination of a large number of product, program, or policy attributes. We evaluated 4 digital information use attributes in the scenarios: the information being used (information type), who is using it (user), the purpose of use (use), and the health condition reflected in the use (health condition). The experimental design included 324 possible scenarios reflecting a full factorial design of 9 information types, 3 users, 3 uses, and 4 health conditions (9 × 3 × 3 × 4 = 324). The survey instrument was adapted from a prior instrument using conjoint analysis to assess consumer privacy preferences related to reusing information obtained from electronic health records (EHRs).^27^ The conjoint attributes and levels were selected based on qualitative interviews with consumers and subject matter experts.^3,28^ We conducted interviews^29^ to evaluate the survey instrument for clarity and participant comprehension prior to administration.

Participants were presented with a brief introduction to the topic of digital health data reuse (eAppendix 1 in the Supplement). The introduction described that there are many sources of digital health information, this information has a broad range of applications, and that it is often possible to identify individuals from the data they leave behind. They were then asked to evaluate 15 scenarios (ie, profiles) randomly selected from the 324 total. Participants rated each scenario on a 5-point scale assessing their willingness to share their information: 1 represented definitely would share, and 5 represented definitely would not share. We reversed the scale in analyses for interpretability of results.

Scenarios were constructed (eAppendix 2 in the Supplement) using 3 different users of the participant’s data: a university hospital, a pharmaceutical company, and a digital technology company. The 3 possible uses of data included research, health care quality improvement, and marketing. The 4 health conditions included cancer, diabetes, depression, and COVID-19. COVID-19 was added because of its relevance during the time of the survey, and we hypothesized participants might be motivated to share data to control its spread.

The information types were chosen to reflect a range relevant for health, including personal spending and finances through banks and credit cards, places visited via public cameras, communication via social media, internet searches via search engines, places visited via smartphone applications, health via EHRs, purchases via online retail, genetic information via consumer genetic testing companies, and walking via smartphone applications.

### Statistical Analysis

Panel recruitment rates were calculated by Ipsos KnowledgePanel and are reported elsewhere.^20^ Study-specific completion rates (percentage of those invited to participate that responded) are reported in the Results section as recommended for online probability samples.^30^

In conjoint analysis, a profile is described in terms of attributes, with each attribute taking specific values. The overall attractiveness of a profile is based on how much each of these parts is worth to the decision-maker (ie, the part-worth utilities). In this study, the part-worth utilities for each level of each conjoint attribute were computed using a generalized estimating equation (GEE) model under a Gaussian distribution and identity link and assuming an independent working correlation structure with robust, empirical SEs. In these models, positive coefficients represent more favored levels and negative coefficients represent less favored levels as compared with a baseline level for each attribute. For each attribute, the range of the part-worths (ie, maximum minus minimum) provides a signal of how important that attribute is in determining the attractiveness of the profiles. To facilitate a comparison of the importance across attributes, the range of each attribute is normalized by the sum of the ranges across attributes. These percentage importance weights were calculated for each attribute. Poststratification weights provided by Ipsos were used in all analyses; these weights ensure that the sample is representative of the US population (by comparing the sample with population benchmarks from the Current Population Survey), reflect study-specific design (eg, oversampling of certain populations), and account for differential distributions of participant and nonparticipant characteristics. All hypothesis tests were 2-sided; *P* < .05 was considered statistically significant.

In addition, a latent class analysis was used to identify and describe clusters of participants who responded similarly to conjoint profiles. The final model (3 clusters) was selected based on Akaike information criterion (AIC) and the Bayesian information criterion (BIC) in conjunction with pragmatic interpretation of results. We assigned 337 participants to their own cluster prior to running the latent class analysis because there was no variability in their responses to all conjoint scenarios (ie, they answered definitely would not share to all). Latent class analysis was conducted in R version 4.0.5 using the flexmix package (R Project for Statistical Computing). All other analyses were conducted in Stata version 16 (StataCorp). Descriptive statistics for the 3 clusters and the excluded group were calculated and compared using χ^2^ statistics.

## Results

We surveyed 6284 potential participants; 3543 responded (56%). Table 1 describes the characteristics of the survey respondents. A total of 1862 participants (53%) were female, 759 (21%) identified as Black, 834 (24%) identified as Hispanic, and 1274 (36%) were 60 years or older.

**Table 1.** Participant Demographic Characteristics

Characteristic	Participants, No. (%) (N = 3543)
Gender	
Male	1681 (47)
Female	1862 (53)
Race	
White	2527 (71)
Black	759 (21)
≥2 Races	109 (3)
Other^a^	148 (4)
Ethnicity	
Hispanic	834 (24)
Non-Hispanic	2709 (76)
Age, y	
18-29	428 (12)
30-44	840 (24)
45-59	1001 (28)
≥60	1274 (36)
Household income, $	
≤24 999	477 (13)
25 000-49 999	673 (19)
50 000-99 999	1166 (33)
≥100 000	1227 (35)
Region	
Northeast	580 (16)
Midwest	668 (19)
South	1443 (41)
West	852 (24)
Metropolitan or nonmetropolitan^b^	
Metropolitan	3154 (89)
Nonmetropolitan	389 (11)
Health status, No./total No. (%)	
Excellent, very good, or good	3015/3531 (85)
Fair or poor	516/3531 (15)
Political ideology, No./total No. (%)	
Liberal	1046/3480 (30)
Moderate	1298/3480 (37)
Conservative	1136/3480 (33)

^a^ Other includes American Indian, Asian, and Hawaiian and Pacific Islander.

^b^ Individuals residing in a metropolitan statistical area are defined as metropolitan; those not living in a metropolitan statistical area are defined as nonmetropolitan.

### Willingness to Share Personal Digital Data

Results from the conjoint experiment using a homogenous model are summarized in Table 2. The relative importance (importance weight on a 0%-100% scale) was greatest for information type (59.1%) followed by user (17.3%), use (12.3%), and disease (11.3%).

**Table 2.** Part-Worth Utilities From Conjoint Scenarios^a^

Attribute	Coefficient (95% CI)	*P* value
User		
Pharmaceutical company	−0.19 (−0.22 to −0.16)	<.001
Digital technology company	−0.23 (−0.26 to −0.20)	<.001
University hospital	[Reference]	NA
Information type		
Places you visit via applications on your phone	0.00 (−0.06 to 0.05)	.94
Places you visit via public cameras	−0.28 (−0.33 to −0.22)	<.001
Walking via applications on phone	0.22 (0.17 to 0.28)	<.001
Internet searches via search engines	−0.11 (−0.17 to −0.06)	<.001
Purchases via online retail	0.01 (−0.04 to 0.07)	.63
Genetic info via genetic testing companies	0.06 (0.00 to 0.12)	.05
Communication via social media	−0.20 (−0.26 to −0.15)	<.001
Spending or finances via banks and credit cards	−0.56 (−0.62 to −0.50)	<.001
Health via EHR	[Reference]	NA
Use		
Clinical care	−0.06 (−0.09 to −0.03)	<.001
Marketing	−0.16 (−0.19 to −0.13)	<.001
Research	[Reference]	NA
Disease		
Diabetes	−0.05 (−0.08 to −0.01)	.008
Depression	−0.09 (−0.13 to −0.06)	<.001
COVID-19	0.05 (0.02 to 0.09)	.003
Cancer	[Reference]	NA

Abbreviations: EHR, electronic health care record; NA, not applicable.

^a^ Part-worth utilities from linear generalized estimating equation model.

Model coefficients in Table 2 represent differences in the primary outcome measure, ie, willingness to share personal digital information (range, 1-5, with 1 indicating definitely would not share and 5 indicating definitely would share). In comparison with health information from personal health care records (ie, EHRs), participants were less willing to share information about their finances from financial institutions (coefficient, −0.56; 95% CI, −0.62 to −0.50), places they visit from public cameras (coefficient, −0.28; 95% CI, −0.33 to −0.22), communication with other people on social media (coefficient, −0.20; 95% CI, −0.26 to −0.15), and their search history from internet search engines (coefficient, −0.11; 95% CI, −0.17 to −0.06). Participants were more willing to share information about their walking activity from applications on their phone (coefficient, 0.22; 95% CI, 0.17-0.28) in comparison with information from their EHR. There were no differences in participants’ willingness to share their genetic information from consumer genetic testing companies, retail purchase history from online retail stores (past purchases), or location information about places they visit from their mobile phone compared with their personal EHR data.

Compared with a university hospital, participants were less willing to share their digital information with a pharmaceutical company or a digital technology company. Compared with research uses, they were less willing to share their information when it would be used for health care quality improvement or for marketing. Differences by disease were generally small, although participants were somewhat less willing to share digital information related to depression and diabetes compared with cancer and more willing to share digital information related to COVID-19.

### Latent Class Analysis

Table 3 reports the results from a latent class analysis; 337 participants were universally opposed to sharing their digital data under any scenario and so were assigned to their own cluster. This cluster is not reported in Table 3, as they were not formally included in the latent class analysis because they revealed no heterogeneity in response to varying conjoint attributes.

**Table 3.** Latent Class Analysis

Attribute	Averse (n = 1155)		Uncertain (n = 1589)		Agreeable (n = 462)	
	Coefficient (95% CI)	*P* value	Coefficient (95% CI)	*P* value	Coefficient (95% CI)	*P* value
Intercept	2.02 (1.97 to 2.07)	<.001	3.29 (3.23 to 3.34)	<.001	4.28 (4.21 to 4.35)	<.001
User						
Pharmaceutical company	−0.18 (−0.21 to −0.15)	<.001	−0.22 (0.25 to −0.19)	<.001	−0.09 (−0.12 to −0.04)	<.001
Digital technology company	−0.20 (−0.23 to −0.17)	<.001	−0.32 (−0.36 to −0.29)	<.001	−0.07 (−0.11 to −0.02)	.004
University hospital	[Reference]	NA	[Reference]	NA	[Reference]	NA
Information type						
Places you visit via applications on your phone	−0.06 (−0.12 to −0.01)	.02	0.03 (−0.03 to 0.09)	.35	0.06 (−0.01 to 0.14)	.10
Places you visit via public cameras	−0.25(−0.31 to −0.20)	<.001	−0.34 (−0.39 to −0.28)	<.001	−0.09 (−0.17 to −0.02)	.02
Walking via applications on phone	0.14 (0.09 to 0.19)	<.001	0.34 (0.28 to 0.40)	<.001	0.11 (0.03 to 0.18)	.004
Internet searches via search engines	−0.20 (−0.24 to −0.14)	<.001	−0.09 (−0.15 to −0.03)	.002	0.02 (−0.05 to 0.10)	.55
Purchases via online retail	−0.08 (−0.13 to −0.03)	.003	0.03 (−0.03 to 0.09)	.33	0.04 (−0.04 to 0.12)	.30
Genetic info via direct-to-consumer genetic testing companies	0.00 (−0.05 to 0.06)	.90	0.13 (0.08 to 0.19)	<.001	0.06 (−0.02 to 0.13)	.12
Communication via social media	−0.21(−0.26 to −0.16)	<.001	−0.21 (−0.27 to −0.15)	<.001	−0.02 (−0.10 to 0.06)	.57
Spending or finances via banks and credit cards	−0.46 (−0.51 to −0.40)	<.001	−0.85 (−0.91 to −0.79)	<.001	−0.32 (−0.40 to −0.24)	<.001
Health care records via EHRs	[Reference]	NA	[Reference]	NA	[Reference]	NA
Use						
Clinical care	−0.07 (−0.10 to −0.04)	<.001	−0.10 (−0.13 to −0.06)	<.001	0.01 (−0.03 to 0.06)	.51
Marketing	−0.15(−0.18 to −0.12)	<.001	−0.19 (−0.23 to −0.16)	<.001	−0.04 (−0.08 to 0.01)	.11
Research	[Reference]	NA	[Reference]	NA	[Reference]	NA
Disease						
Diabetes	−0.07 (−0.11 to −0.04)	<.001	−0.08 (−0.12 to −0.04)	<.001	0.00 (−0.05 to 0.05)	.92
Depression	−0.12 (−0.16 to −0.09)	<.001	−0.14 (−0.18 to −0.11)	<.001	−0.08 (−0.13 to −0.03)	.003
COVID-19	0.05 (0.02 to 0.08)	.005	0.05 (0.02 to 0.09)	.006	0.03 (−0.02 to 0.09)	.18
Cancer	[Reference]	NA	[Reference]	NA	[Reference]	NA
Intercept only model						
No.	1116	NA	1616	NA	474	NA
Intercept	1.64 (1.62 to 1.65)	<.001	2.84 (2.81 to 2.86)	<.001	4.18 (4.16 to 4.21)	<.001

Abbreviations: EHR, electronic health care record; NA, not applicable.

The most notable variation across the 3 clusters from the latent class analysis was in the model intercept, reflecting that the 3 derived clusters differed most in their general willingness to share information and that less difference was found among their views about specific uses, users, information types, or disease conditions. For that reason, the clusters were labeled as an averse cluster (1155 respondents [32.5%]), an uncertain cluster (1589 [44.8%]), and an agreeable cluster (462 [13.0%]), to which might be added a never cluster (337 [9.5%]), representing the participants unwilling to share any information.

The 3 clusters exhibited similar trends across the specific sharing scenarios. Among all 3 clusters, the importance weight was greatest for information type (averse: 53.1%; uncertain: 62.5%; agreeable: 63.1%). Individuals were least likely to share data reflecting personal finances (averse: coefficient, −0.46; 95% CI, −0.51 to −0.40; uncertain: coefficient, −0.85; 95% CI, −0.91 to −0.79; agreeable: coefficient, −0.32; 95% CI, −0.40 to −0.24) and places they visit from public cameras (averse: coefficient, −0.25; 95% CI, −0.31 to −0.20; uncertain: coefficient, −0.34; 95% CI, −0.39 to −0.28; agreeable: coefficient, −0.09; 95% CI, −0.17 to −0.02). Individuals were most likely to share data that reflected on their physical activity from apps on their phone (averse: coefficient, 0.14; 95% CI, 0.09-0.19; uncertain: coefficient, 0.34; 95% CI, 0.28-0.40; agreeable: coefficient, 0.11; 95% CI, 0.03-0.18). The Figure illustrates variation across 8 possible scenarios for the 3 clusters. Across-group variation was large, while within-group variation was largest for the uncertain group. For example, the agreeable group was most willing to share information from walking apps with a university hospital for research purposes (mean, 4.39; 95% CI, 4.32-4.46) compared with the uncertain (mean, 3.63; 95% CI, 3.58-3.67) and averse (mean, 2.16; 95% CI, 2.10-2.21) groups. Within the uncertain group, individuals were more willing to share information from walking apps with a university hospital for research purposes (mean, 3.63; 95% CI, 3.58-3.67) compared with financial information (mean, 2.44; 95% CI, 2.39-2.50). 

**Figure.**
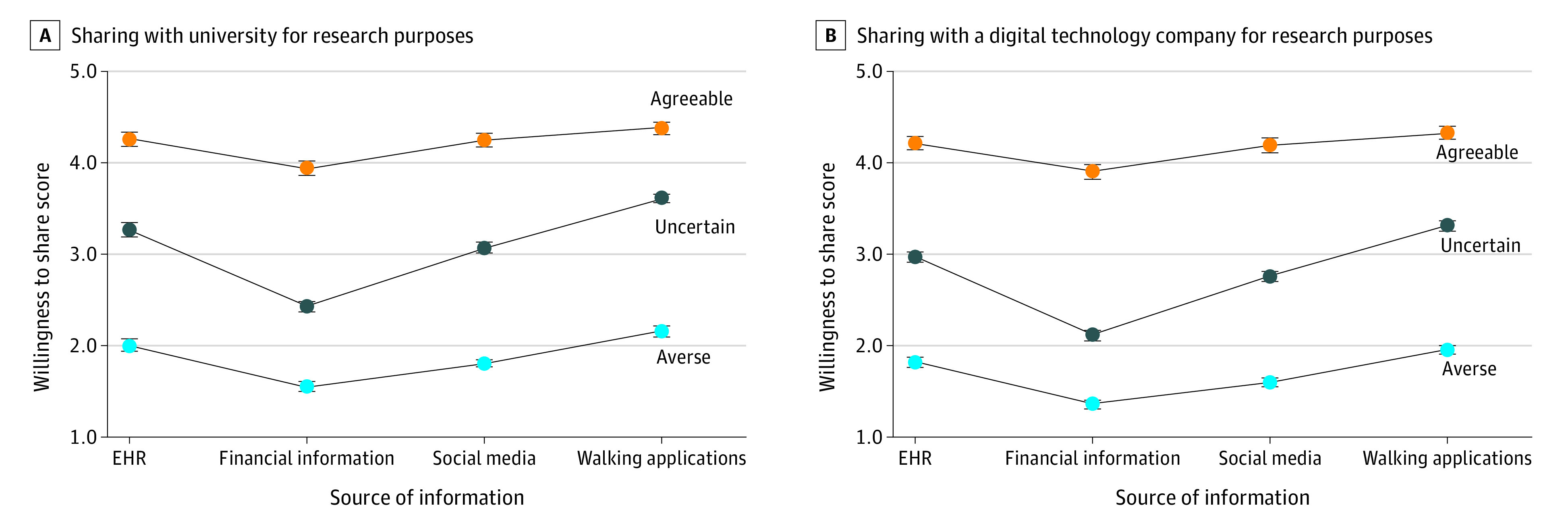
Variation in Willingness to Share Digital Information for Research by Subgroup Point estimates reflect participants’ willingness to share for a subset of scenarios from the conjoint experiment (willingness to share evaluated on a 1-5 scale). Each scenario had approximately 591 participants randomized to that scenario (the number of respondents ranged from 569 to 606). Panel A represents a university hospital and Panel B represents a digital technology company as the user of the data.

Given the large variation in model intercepts in the latent class analysis, we also show results in Table 3 of an intercept-only model. This model reflects the mean ratings of respondents without regard for the assigned conjoint scenarios. Latent class analysis again results in 3 groups with similar numbers of respondents and intercepts to the full model (averse: mean rating, 1.64; 95% CI 1.62-1.65; uncertain: mean rating, 2.84, 95% CI, 2.81-2.86; agreeable: mean rating, 4.18, 95% CI, 4.16-4.21).

### Characteristics of Clusters

Table 4 shows the characteristics of 4 subgroups: the 3 clusters from the latent class analysis and the fourth group that was created given their universal opposition to sharing information under any situation. White participants and those with higher incomes were more likely to be in a more privacy-concerned cluster (averse and never clusters) than those with lower incomes and racial and ethnic minority participants. Those in fair or poor health were less likely to be in a more privacy-concerned cluster. Politically conservative respondents were more likely to be in a privacy-concerned cluster, particularly the group that was universally opposed to sharing (comprising 49.5% of that group [162 respondents] vs 32.6% of the overall study population [1136 respondents]).

**Table 4.** Characteristics of Subgroups

Characteristic	Overall (N = 3543)	Never (n = 337)^a^	Averse (n = 1155)	Uncertain (n = 1589)	Agreeable (n = 462)	*P* value
Gender						
Male	1681 (47.4)	168 (49.9)	551 (47.7)	744 (46.8)	218 (47.2)	.78
Female	1862 (52.6)	169 (50.1)	604 (52.3)	845 (53.2)	244 (52.8)	
Race						
White	2527 (71.3)	253 (75.1)	867 (75.1)	1112 (70.0)	295 (63.9)	.002
Black	759 (21.4)	62 (18.4)	211 (18.3)	357 (22.5)	129 (27.9)	
≥2 Races	109 (3.1)	10 (3.0)	29 (2.5)	54 (3.4)	16 (3.5)	
Other^b^	148 (4.2)	12 (3.6)	48 (4.2)	66 (4.2)	22 (4.8)	
Ethnicity						
Hispanic	834 (23.5)	59 (17.5)	224 (19.4)	398 (25.0)	153 (33.1)	<.001
Non-Hispanic	2709 (76.5)	278 (82.5)	931 (80.6)	1191 (75.0)	309 (66.9)	
Age, y						
18-29	428 (12.1)	36 (10.7)	121 (10.5)	215 (13.5)	56 (12.1)	.09
30-44	840 (23.7)	68 (20.2)	263 (22.8)	388 (24.4)	121 (26.2)	
45-59	1001 (28.3)	109 (32.3)	336 (29.1)	426 (26.8)	130 (28.1)	
≥60	1274 (36.0)	124 (36.8)	435 (37.7)	560 (35.2)	155 (33.5)	
Household income, $						
≤24 999	477 (13.5)	44 (13.1)	125 (10.8)	230 (14.5)	78 (16.9)	<.001
25 000-49 999	673 (19.0)	51 (15.1)	178 (15.4)	330 (20.8)	114 (24.7)	
50 000-99 999	1166 (32.9)	118 (35.0)	402 (34.8)	509 (32.0)	137 (29.7)	
≥100 000	1227 (34.6)	124 (36.8)	450 (39.0)	520 (32.7)	133 (28.8)	
Region						
Northeast	580 (16.4)	55 (16.3)	183 (15.8)	280 (17.6)	62 (13.4)	.06
Midwest	668 (18.9)	70 (20.8)	232 (20.1)	297 (18.7)	69 (14.9)	
South	1443 (40.7)	126 (37.4)	455 (39.4)	652 (41.0)	210 (45.5)	
West	852 (24.0)	86 (25.5)	285 (24.7)	360 (22.7)	121 (26.2)	
Metropolitan or nonmetropolitan^c^						
Metropolitan	3154 (89.0)	288 (85.5)	1017 (88.1)	1426 (89.7)	423 (91.6)	.03
Nonmetropolitan	389 (11.0)	49 (14.5)	138 (11.9)	163 (10.3)	39 (8.4)	
Health status						
Excellent, very good, or good	3015 (85.4)	289 (86.3)	1020 (88.7)	1316 (83.0)	390 (84.6)	.001
Fair or poor	516 (14.6)	46 (13.7)	130 (11.3)	269 (17.0)	71 (15.4)	
Political ideology						
Liberal	1046 (30.1)	67 (20.5)	330 (29.1)	509 (32.6)	140 (30.8)	<.001
Moderate	1298 (37.3)	98 (30.0)	422 (37.2)	591 (37.8)	187 (41.1)	
Conservative	1136 (32.6)	162 (49.5)	383 (33.7)	463 (29.6)	128 (28.1)	

^a^ Assigned to cluster prior to latent class analysis because there was no variability in their responses to all conjoint scenarios (answered definitely would not share to all).

^b^ Other includes American Indian, Asian, and Hawaiian and Pacific Islander.

^c^ Individuals residing in a metropolitan statistical area are defined as metropolitan; those not living in a metropolitan statistical area are defined as nonmetropolitan.

## Discussion

This study has 3 main findings. First, consumers generally have views about privacy that shift only modestly based on context. Just more than half of respondents (55%) had preferences to share or not share their information that were largely independent of context: 10% were universally opposed to all sharing, 33% were opposed to most sharing, and 13% were in favor of most sharing. The remainder (45%) revealed preferences more responsive to the context of information reuse. Our findings are consistent with results from a 2019 survey by the Pew Research Center,^31^ which found that many respondents were skeptical about sharing personal data but that support varied depending on the use (support for a range of health and nonhealth digital reuse scenarios varied between 25% and 49%, with 18%-27% of respondents with neutral views).

Second, the 4 privacy subgroups varied in their demographic composition. White and higher-income populations were more likely to be in a privacy-concerned subgroup (never or averse subgroup) than individuals from racial and ethnic minority populations or those from lower-income households. Prior research suggests that racial and ethnic minority populations and individuals from low-income households have greater concerns about digital privacy; however, these concerns often stem from concerns about specific harms from incidents related to identity theft or government surveillance.^32,33^ Our study was more focused on nongovernmental uses of digital health information including nonprofit research uses as well as programs focused on health care quality or commercial health-related activities.

Third, although many respondents held strong views about whether they wished to share or not share their data, the specific context related to information reuse is still an important factor. Financial information, information from passive monitoring of location (from cameras), and monitoring chatter on social communication were evaluated as more sensitive. In contrast, information that currently has greater protection (ie, EHR data) or areas that have historically drawn the attention of ethicists and policy makers (ie, genetic data) were evaluated as less sensitive. With the lines between health and nonhealth information blurred more than ever, the traditional boundaries around different types of information may no longer be relevant.

### Implications

The proliferation of digital health data from a wide range of sources creates many opportunities to develop programs and tools to improve health. Our study shows that many consumers—when given a choice—are reluctant to share their digital health information. Rather than context driving preferences, many consumers seemed to have strong views about sharing or not sharing information regardless of the specific use case scenario. The key question is whether enhanced privacy protections would increase trust and support for socially beneficial uses. Prior research suggests that when protections are perceived to be stronger (ie, consumers have greater control), consumers have fewer privacy concerns.^34^ The current lack of protections may hinder consumer support for health programs powered by consumer digital data and data science.

### Limitations

Our study has limitations. First, because of the cross-sectional design, the findings represent a moment in time when the survey was administered (July 2020). Second, the findings represent the results of rating hypothetical scenarios rather than actual decisions. However, conjoint analysis is a rigorous and validated approach to measure preferences and predict real-world decisions.^25,26^ Third, as with all survey research, nonresponse bias is a concern. However, the experimental design allows for strong internal validity from the conjoint experiment. In addition, the sample is drawn from a nationally representative panel population, and the participation rate among sampled individuals was high and similar to other published studies.^27,35^

## Conclusions

In this national survey study using conjoint analysis, we found that US adults held privacy views about their personal digital data that were partially informed by the context of use (ie, the specific use case scenario). However, these views were largely associated with participants’ underlying preferences about digital privacy overall.

## Notes

Supplement. **Appendix 1.** Conjoint Survey Instrument **Appendix 2.** Conjoint Design Table
